# Sports-based recreation as a means to address social inequity in health: why, when, where, who, what, and how

**DOI:** 10.1186/s12889-019-7428-3

**Published:** 2019-08-09

**Authors:** Peter Elsborg, Glen Nielsen, Charlotte D. Klinker, Paulina S. Melby, Julie H. Christensen, Peter Bentsen

**Affiliations:** 1 Health Promotion Research, Steno Diabetes Center Copenhagen, Gentofte, Denmark; 2 Department of Nutrition, Exercise and Sports, University of Copenhagen, Copenhagen, Denmark; 3 Department of Geosciences and Natural Resource Management, University of Copenhagen, Copenhagen, Denmark

**Keywords:** Disadvantaged neighborhoods, Health, Low socioeconomic status, Noncommunicable diseases, Recreation, Social inequity in health, Youth

## Abstract

The rising global burden of noncommunicable diseases (NCDs) among people with low socioeconomic status (SES) has heightened awareness of the need for primary prevention programs in low-SES neighborhoods. Social inequity in health is apparent in mental, social and physical aspects of health among people living in low-SES neighborhoods. Viewing this problem from a life course perspective and adopting a vulnerable population approach points to the importance of inducing sustainable health behavior changes in children and young people living in low-SES neighborhoods. One important factor in lowering the risk of many NCDs while improving mental health is the promotion of physical activity (PA). In this paper, we argue that lowering the risk of many NCDs and improving mental health is best achieved through setting-based programs that facilitate long-term PA behavior changes in children and adolescents living in marginalized neighborhoods. Empirical evidence indicates that extrinsic motives for participating in physical activities, such as improving health, are insufficient when long-term participation is the goal. Therefore, we argue that interventions with the aim of affecting long-term PA in low-SES neighborhoods and thereby reducing social inequities in health should include activities that aim to create more intrinsic and autonomous motivations by building on more broad and positive understandings of health and participation. Here, we advocate that sports-based recreation (SR) holds several advantages. If implemented well, SR has the potential to be a health-promoting activity that is meaningful and motivating in itself and that involves physiological health-promoting aspects (e.g., PA), a social aspect (e.g., positive relations with others), and a psychological aspect (e.g., positive experiences of oneself). Further, we suggest four practicalities that should be considered when conducting interventions: the cost of participating, the location, the facilities required, and the suitability of the SR activities.

## Background

Noncommunicable diseases (NCDs), such as diabetes, cancer, and cardiovascular and chronic respiratory diseases, constitute a major and increasing public health challenge in both high-, low- and middle-income countries [1]. Across and within countries, there is a social gradient in health: the lower in the social hierarchy you are located, the greater your risk for a number of preventable diseases and the lower your expected life span [1, 2]. This social inequity in health is just as apparent in the mental and social aspects of health as in the more physical aspects. Living in socioeconomically disadvantaged neighborhoods, neighborhoods with a high percentage of people with little financial and educational resources, has been shown to have a negative impact on mental and social health. These inequalities in health and well-being and differences between subpopulations in society can be reduced and should therefore be addressed with preventive measures and interventions [1]. One important factor in lowering the risk of many NCDs and some mental health challenges is the promotion of physical activity (PA) [3].

Individuals who are physically inactive have an increased risk of developing a number of preventable lifestyle-related chronic diseases, e.g., diabetes [3]. The recent Lancet series on PA estimated that physical inactivity costs health-care systems more than $50 billion worldwide annually [4]. Recent evidence suggests that the average amount of PA among children and young people is too low, with only one in five adolescents worldwide meeting the international recommendations of at least 1 hour of moderate to vigorous PA a day [5]. In a newly published international large-scale study based on 49 different countries this was confirmed [6]. Girls have a significantly lower level of PA than boys throughout their adolescent years [5, 7], and for both genders, PA levels decrease with age [8]. This situation is serious, as research has shown that unhealthy habits established early in life have a greater negative effect on the body because the body is not yet developed and because these habits tend to be maintained in adulthood [7]. At the same time, there is a significant social inequity in relation to PA engagement. For example, a Danish study showed that girls with ethnic minority backgrounds are less active than girls with only Danish backgrounds [9]. Recently data from the international Health Behaviour in School-aged Children (HBSC) study showed that this is the case across nations [10].

In this paper, we first argue for the value of adopting a life course, a setting and a vulnerable population approach to reducing inequity in health. Second, by combining these health promotion approaches, we find that certain types of physical activities hold special potential as a solution to the inequity problem above and beyond other types of PA. In the following section the Life Course Approach, the Setting Approach and the Vulnerable Populations Approach to health promotion will be described.

### When, where and who?

#### Life course, setting and vulnerable population approaches to health promotion

**Fig. 1:**
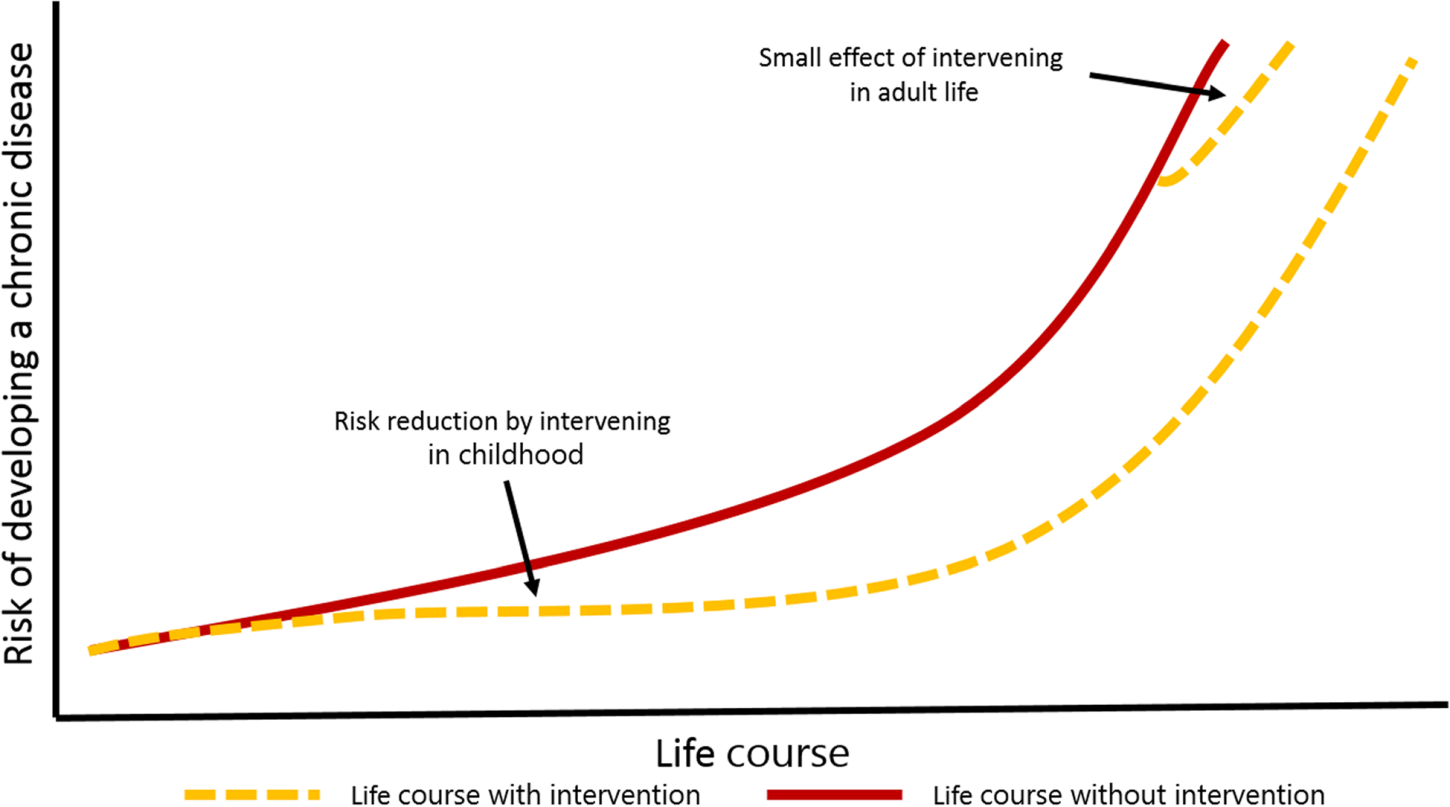
The life course approach. The illustration inspired by Hanson & Gluckman (2011) shows that interventions in childhood are more effective through the life course compared to interventions in adulthood. Permission to reproduce this figure was given by the original authors

In this section we will present three health promotion approaches and argue for how they in combination illuminate when in individual’s life it is important to intervene, where interventions would be most effective and who the target group should be, when the goal is to reduce social inequity. It has been shown that the risk of developing NCDs is cumulative over the life course [11]. Research has also revealed that a high level of PA over the life course is associated with improved health through the reduction of risk factors for chronic diseases, such as type 2 diabetes and cardiovascular disease [5, 12]. Recent research on interventions aimed at reducing the risk of NCDs has shown that interventions early in life have a higher impact than interventions later in life [11]. Furthermore, the cumulative effect of risk exposure through a life course seems to be particularly strong for low-income groups, as studies show that low-income groups are more susceptible to life style risks such as physical inactivity [13]. As the risk of NCDs is cumulative throughout life, the life course approach calls for interventions to be implemented as early in life as possible (Fig. 1). Recently, it was suggested that not only the first 1000 days but also the subsequent 7000 days (i.e. 22 years of age) of a child’s life are of great significance to health, which means that in addition to interventions during pregnancy and early infancy, school interventions and youth community programs play an important and supplementary role in primary prevention [14]. These findings all point to the benefits of focusing on children and youth in health promotion in general and in health promotion through PA more specifically, especially when addressing social inequalities in health.

Frohlich and Potvin [13] suggested that focusing health promotion efforts on vulnerable populations is necessary for reducing inequalities in health. The authors defined vulnerable populations as *“a subgroup or subpopulation who, because of shared social characteristics, is at higher risk of risks”* [13]. Residents of low socioeconomic status (SES) neighborhoods represent one such subgroup with shared social characteristics. Vulnerable population approaches should supplement population approaches, as described by Rose [15], to reduce inequalities in health and to help avoid a narrow focus on individual risk behavior [13].

A vulnerable population approach to health promotion may be best facilitated in setting-based interventions and initiatives, especially when the target group is socioeconomically disadvantaged and young, as these characteristics limit independent mobility [16]. The setting-based approach is grounded on the insight that health promotion interventions must consider the context in which they intervene since the context affects the intervention and is an influential determinant in itself [17, 18]. To influence behavioral risk factors, such as low PA, prevent and reduce NCDs and reduce social inequity in health, we need to reach children and young people in settings and communities with a high risk of developing NCDs.

**Fig. 2:**
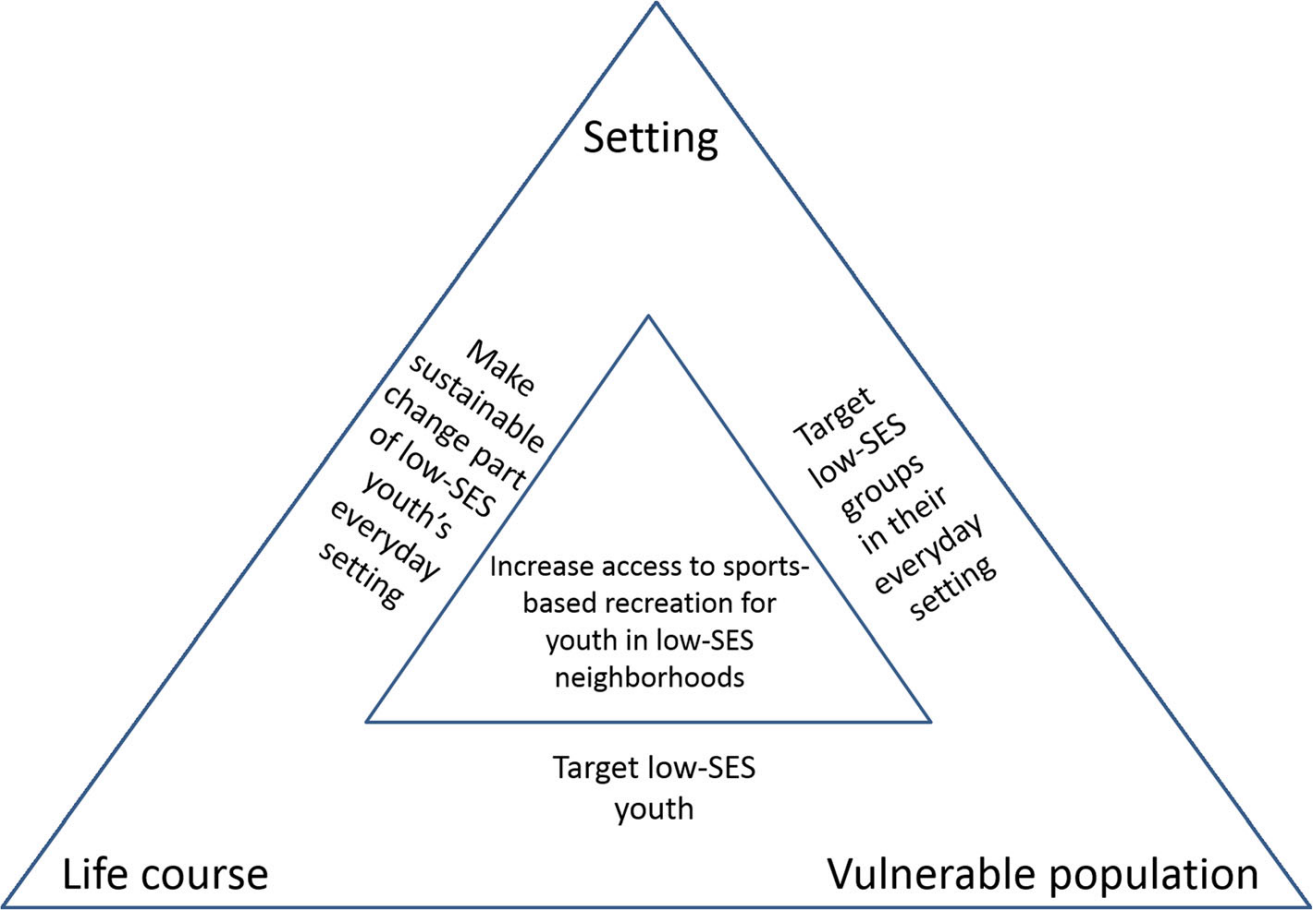
The setting, life course and vulnerable approaches triangle. The triangle illustrates how a vulnerable population, a life course and a setting approach are related and how in combination, they point to that interventions should target increasing participation in sports-based recreation among children and youth in low-SES neighborhoods

In this debate paper, we argue that promoting sports-based recreation (SR) activities for children and youth in low-SES neighborhoods is an one prolific way to combine a vulnerable population, a life course and a setting-based approach while preventing and addressing social inequities in NCDs. This is illustrated in Fig. 2. Arguments for and evidence that supports our argument are presented and discussed below. In this paper, SR is understood as leisure-time involvement in activities that include PA and are inspired by one or more types of sports. SR can be offered in formalized organizations and clubs or in more spontaneous, informal self-organized settings. Even though we argue for increasing SR participation as a means to reduce inequity in health, it is important to note that we also argue that the purpose for the participants performing SR should be primarily inherent in the activity.

### What?

#### The potential of sports-based recreation as health promotion for marginalized youth

While recognizing the large body of literature originating in a sport for development perspective [19] as important, the potentials of SR are also highly relevant within the public health, prevention and health promotion perspective. When combining a life course, a vulnerable population and a setting approach to promote lifelong and sustainable participation in physical activities in order to reduce social inequity in health, it is important to consider which physical activities should be adopted, what elements of the activities are influential, and at what stages in life they are most important and have the greatest potential. Within exercise physiology, many experimental studies have investigated the effect of PA on physiological health outcomes. The focus has mainly been on the effects of individual exercise activities, such as running on a treadmill and cycling on an exercise bike, and it has been shown that these activities have positive effects on physical health factors. However, this does not mean that such activities are the healthiest from a broader health perspective. If the activity is done as a replacement for or is prioritized over more social physical activities such as dancing or playing a team sport, the potential social health effect of being physically active is neglected, and consequently, the collective health effect may be smaller compared to that of an activity that entails both social relations and PA [20].

Distinctions between types of physical activities such as exercise and sports have been described by, e.g., by Khan and colleagues [21], who illustrated this in their Lancet paper inspired by Caspersen, Powell and Christensen’s [22] iconic article on the subject. When comparing different types of physical activities, empirical evidence suggests that in general participation in organized sports is important for most children’s and young people’s engagement in PA, especially their amount of moderate to vigorous PA (MVPA), throughout the life course [21, 23]. Marques, Ekelund and Sardinha [24] showed in a cross-sectional study that the odds of meeting PA recommendations were significantly higher (OR = 1.64) for those who participated in an organized sport compared with those who did not. Accordingly, a longitudinal cohort study found that organized sports participation, measured through systematic text messages to parents, was significantly associated with increased levels of PA. Frequency of weekly participation in sports, especially participation in soccer, increased MVPA [25]. The results from the same cohort study also showed that organized sports participation significantly reduced the risk of developing cardiovascular disease. The researchers also found that team ball sports, such as handball and soccer, had a particularly positive effect on physiological health measures [23]. Khan and colleagues [21] concluded in their article on organized sports participation as a strategy for increasing the health of nations that *“Sport is associated with 20-40% reduction in all cause mortality compared with non-participation”.* However, as previously mentioned, the health effects of participation in sports are not limited to the physical aspects of health. A large systematic review found that participation in sports is associated with increased mental health and concluded that “*Specifically, club-based or team-based sport seems to be associated with improved health outcomes compared to individual activities, due to the social nature of the participation”* [26]*.* In addition, it has been shown that participation in organized sports for youth can have other benefits for the individual and society if the programs are designed in accordance with evidence-based theory on implementation of such interventions [27, 28], such as increases in life skills [29], positive youth development [28, 30], reductions in risky behaviors [31], increased school performance [32], and crime prevention [27].

#### The adherence potential of sports-based activities

From a life course perspective on health, adherence is a vital aspect of PA accumulation. Here, it seems that SR activities are more motivating and have a higher degree of adherence than other types of physical activities [33, 34]. In numerous studies on PA, exercise and sports adherence have been associated with intrinsic motivation as defined by self-determination theory (SDT) [35]. Intrinsic motivation has been found to be crucial for adherence to PA in general. Intrinsic motivation for an activity is the result of the degree to which the basic psychological needs for autonomy, relatedness and competence are satisfied when doing the activity [36, 37]. Here, SR provides extensive opportunities for satisfying the needs for competence and relatedness in particular. The need for competence can be satisfied by the possibility for skill acquisition that is present both tactically and technically in sports, and the need for relatedness can be satisfied by the social nature of sports [38]. Thus, engaging in SR is beneficial for the health of children and youth on a wide spectrum of health-related factors and holds substantial potential for adherence above and beyond other types of PA.

### How?

#### Reducing barriers for engagement in SR for children and youth in low-SES neighborhoods

Ample empirical evidence suggest that social inequity exists within sports participation, where big differences between SES groups has been shown in Australia [39, 40], England [41], Denmark [42], and the Netherlands [43]. A nationwide longitudinal study in Canada found that social inequity was especially apparent when comparing organized sports to an informal context: “*The effects of SES were much stronger for organized sport involvement than for participation in an informal context*” [44]. This result was also found to be the case in Denmark [42], indicating that reducing the barriers to participating in SR among children and youth with a low socioeconomic background should be a prime assignment for interventions with the goal of increasing equality in health and PA. Promoting the informal organization of SR is likely to achieve this goal.

**Fig. 3:**
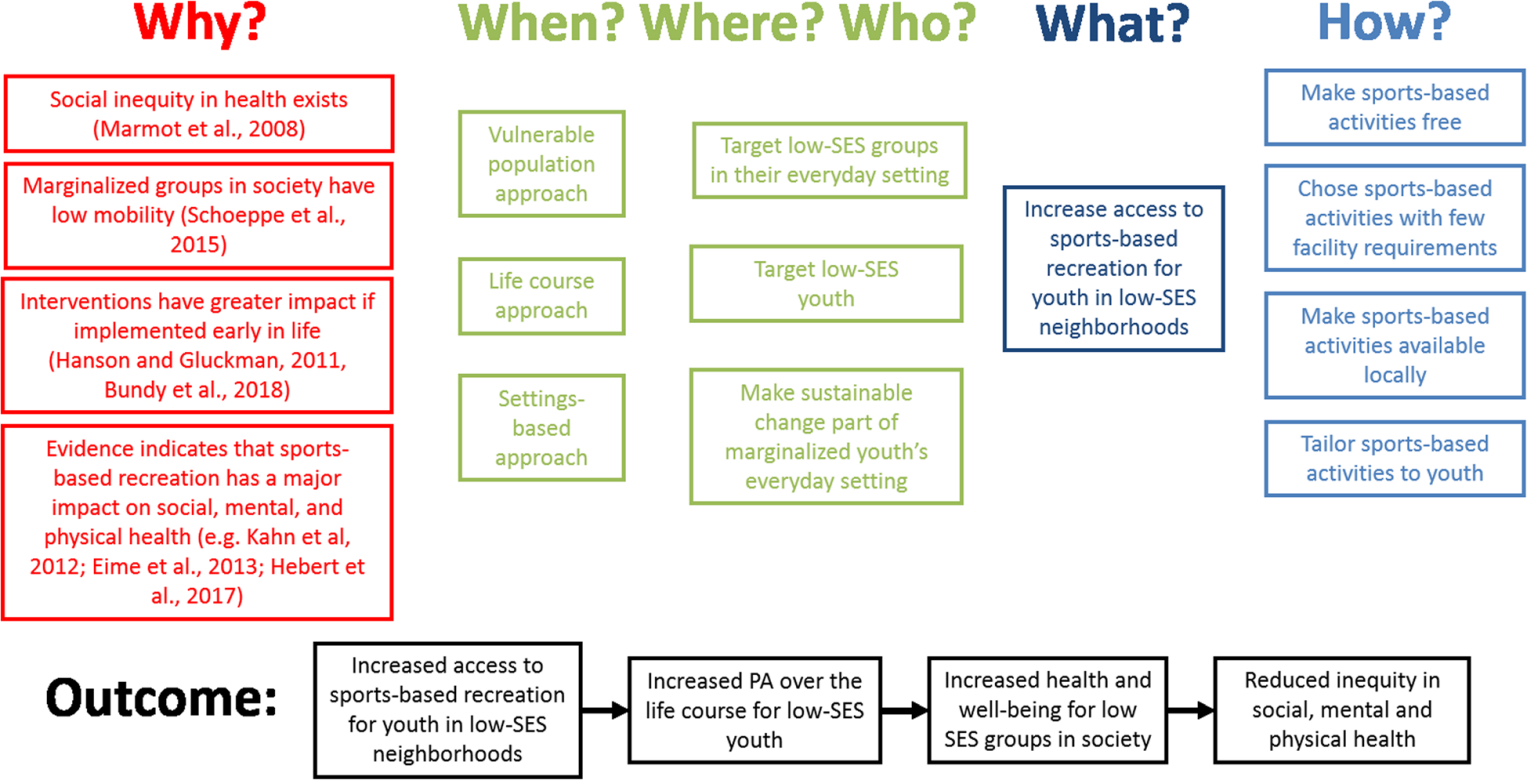
The overall argument—Logic model. Logic model describing why, when, where, who, what and how sports-based recreation can be a means to address social inequity in health

Based on the advantages of the three health-promoting approaches introduced and described earlier, we propose and advocate that four practicalities are central in interventions aimed at reducing barriers for sustainable engagement in SR among children and youth living in low-SES neighborhoods: the cost of participating, the location, the facilities required, and the suitability of the SR activities. These four practicalities are intentional simplistic, meaning that they are easily implemented. However, the practicalities should be understood and used as guideline while recognizing the societal complexities that exists in low SES neighborhoods. A logic model describing how these approaches may work to reduce inequity in health is illustrated in Fig. 3.

Children and youth from families with a low socioeconomic position have fewer resources in terms of finances, means of transportation, social support, and sporting capital, and this has been shown to explain their lower rate of participation in sports [45]. As a result, we propose that interventions with the aim of increasing SR among children and youth living in low-SES neighborhoods need to consider the following practicalities:

1. **Sports-based activities in the intervention should be free of charge**

A barrier to participation in SR for vulnerable groups is the reduced ability of this group to pay the fees levied by organized sport clubs [46]. Studies have suggested that economic incentives influence behavioral choices that are related to participation in sports and exercise [47, 48]. This means that making sport activities or memberships in sport clubs free or almost free for children and youth from low-income families may enable more children and youth to participate. An Australian qualitative study of community strategies undertaken by 62 organizations with the aim of promoting a focus on sports participation in low-SES groups by implementing strategies to address the cost barrier to participation found that when subsidizing costs in different ways, the organizations managed to attract the desired number of participants. However, when user fees were applied after a period, there was a significant reduction in participant numbers [49]. This result demonstrates the potential of creating cost-free opportunities to participate in SR for children and youth belonging to low-SES groups and illustrates the challenge of developing financially sustainable strategies. Another study in the Netherlands found that people with low income had a preference for fee exemptions and discount vouchers that could be redeemed at flexible times and locations [50]. In accordance, studies on a large health promotion effort in the Icelandic capital of Reykjavik showed that a model in which all young people were given a sports pass with a certain amount of money to redeem at one or several sports clubs had several health benefits [51, 52]. Examples of initiatives that facilitate free of charge sports-based recreation targeting low SES groups are the Danish NGO GAME [53] and the organization StreetGames in the UK [54].

2. Sports-based recreation should be possible to do with few or no facilities.

The built environment plays an important role in participation in SR among children and youth living in marginalized neighborhoods because the environment may offer no-cost opportunities for PA close to where they live [55, 56]. As an example, public parks provide opportunities for organized and informal unstructured activities. A qualitative study indicated that children’s levels of PA were positively associated with unstructured activities in public parks [57]. Research has also shown that the availability of sport fields, courts and playgrounds are positively associated with park-based activity in children [58], and observational studies have shown that parks with a sports field have the highest number of park users and are used at higher levels of PA intensity among all age groups [59, 60]. However, study findings regarding the relationships between access and proximity to public parks and recreational settings and PA in youth are inconsistent [4, 61–63]. Thus, it seems that the attributes of parks play a role in the PA behavior of park users and could encourage residents to use park areas to a greater extent. As parks with a sports field are publicly available, they are considered an important setting for SR among children and youth from low-income families who are underrepresented in club-organized sports [45]. These parks are free of cost, and the participants can engage in activity at flexible times. However, it has also been shown that low-SES neighborhoods are significantly less likely to have recreational facilities [64]. Thus, it is important to ensure that SR activities can not only be performed with few or no facilities but also offered in the neighborhoods where children and youth live.

3. Sports-based activities should be located in the local neighborhoods where children and youth live

Children and youth with low SES have a lower participation rate in organized sports [44]. However, they can achieve levels of PA equivalent to other socioeconomic groups based on their participation in PA in other, more informal leisure contexts [45, 65]. To support participation in SR, SR activities should be located in the neighborhoods where low-SES children and youth already engage in most of their PA. Low SES and ethnic minority background has been shown to be associated with lower sporting capital [45, 65], and this has been shown that together with lower economic capital this lower sporting capital can explain (is a mediator for) the lower rates of club-sports participation among children from low SES [45] and ethnic minority backgrounds [65]. As participation in club or organized sports takes place in contexts parents with low sports capital are often unfamiliar with and therefore have little understanding of and trust in it makes it harder for parents with low sporting capital to support and encourage their children’s participation [39, 42]. This can be seen as part of the explanation for lower participation in club or organized sports among children with low SES and ethnic minority backgrounds. Close proximity to facilities for PA is associated with higher levels of vigorous PA in all socioeconomic groups [61]. SR activities should address the enabling resources, dispositions and habits among children with low sociocultural resources for less organized, locally based opportunities for play and PA, which could reduce barriers to participation related to family, cultural and socioeconomic background [60]. This can be seen as part of the explanation for lower participation in club or organized sports among children with low SES and ethnic minority backgrounds. Close proximity to facilities for PA is associated with higher levels of vigorous PA in all socioeconomic groups [66]. SR activities should address the enabling resources, dispositions and habits among children with low sociocultural resources for less organized, locally based opportunities for play and PA, which could reduce barriers to participation related to family, cultural and socioeconomic background [65]. Organized SR that utilizes the facilities in a neighborhood may also lead to new perceptions of neighborhood opportunities for PA among other residents in a low-SES neighborhood if, e.g., local greenspace or other facilities are used for SR in new, different and unexpected ways. Depending on the surroundings, only the imagination limits the possibilities, however examples could be using trees/bushes/bins as goals in soccer, practicing basketball passing in places with uneven ground or using a parking lot for a dancing lesson.

4. Sports-based activities should be tailored to the target group

As physical activity engagement often is under prioritized, it is important to ensure that the sports-based activities, like other health promotion efforts, should be attractive and relevant to the intended target group. In this regard, participatory approaches that engage the target group in defining key aspects of the program are an important step [13]. Accordingly, a systematic review and meta-analysis recently concluded that solid evidence suggests that community engagement interventions have a positive impact on various health factors [67]. When trying to develop locally adapted and accessible activities, children, youth and families should be considered important advisors. Tailoring activities to the young target group may involve identifying activities that are engaging and fun in the setting (this is probably context-dependent), developing age-appropriate activities, ensuring parents’ trust and support, and creating a safe environment for children and youth to thrive.

One way of tailoring an intervention to involve the target group has been suggested in a review to be by adopting a peer-to-peer or a peer support strategy [68]. Although this review was mostly based on interventions targeted adults, the evidence also suggested that this type of strategy was successful in reaching and motivating young people. It also has the potential to develop social capital and knowledge resources in the community, which may result in longer-lasting impacts compared to intervening only from outside the community.

## Conclusion

The rising global burden of NCDs, which are most prominent among people living in low-SES neighborhoods, has heightened awareness regarding the increasing social gradient in health and the need for primary risk prevention programs in low-SES neighborhoods. When adopting a combination of a life course, a setting and a vulnerable population approach to health promotion, we suggest that a feasible solution involves developing health promotion programs that facilitate better conditions for lifelong behavior changes in children and adolescents in low-SES neighborhoods. It has been shown that engaging in SR is beneficial for children and youth’s health on a wide spectrum of health-related factors and offers substantial adherence potential above and beyond other types of PA. Therefore, we argue that health promotion programs based on local, informal SR activities may be an efficient strategy to enhance lifelong engagement in PA among individuals with low SES and thereby affect other important outcomes such as physical, social and mental health. Further, we advocate for considering the cost of participating, the location, the facilities required, and the suitability of the SR activity when developing health promotion programs. Finally, we argue for adaption to the local context by involving the participants and the local community in the organization of the activities, e.g., by using a peer-to-peer approach.

## Data Availability

Not applicable.

## Abbreviations

MVPA: Moderate to vigorous physical activity
NCD: Noncommunicable diseases
OR: Odds ratio
PA: Physical activity
SES: Socioeconomic status
SR: Sports-based recreation
